# Quantitative Evaluations of Geometrical Distortion Corrections in Cortical Surface‐Based Analysis of High‐Resolution Functional MRI Data at 7T

**DOI:** 10.1002/jmri.27420

**Published:** 2020-11-05

**Authors:** Tetsuya Yamamoto, Masaki Fukunaga, Sho K. Sugawara, Yuki H. Hamano, Norihiro Sadato

**Affiliations:** ^1^ Department of System Neuroscience Division of Cerebral Integration, National Institute for Physiological Sciences Okazaki Japan; ^2^ Department of Physiological Sciences, School of Life Science The Graduate School for Advanced Studies (SOKENDAI) Hayama Japan; ^3^ Neural Prosthesis Project, Department of Dementia and Higher Brain Function Tokyo Metropolitan Institute of Medical Science Tokyo Japan

**Keywords:** geometrical distortion, B_0_ inhomogeneity, gradient nonlinearity, ultra‐high field MRI, cortical surface‐based analysis

## Abstract

**Background:**

Although 7T functional MRI (fMRI) provides better signal‐to‐noise ratio and higher spatial resolution than 3T fMRI, geometric distortions become more challenging because fMRI is more susceptible to distortions than structural MRI. Accurate alignment of 7T fMRI to structural MRI data is critical for precise cortical surface‐based analysis.

**Purpose:**

To quantify the effectiveness of distortion corrections of 7T fMRI data.

**Study Type:**

Prospective.

**Subjects:**

Fifteen healthy individuals aged 19–26 years (mean: 21.9 years).

**Field Strength/Sequence:**

Multiband gradient‐echo echo‐planar imaging sequence at 7T; 3D T_1_/T_2_‐weighted sequences (magnetization prepared rapid acquisition with gradient echo [MPRAGE] and sampling perfection with application optimized contrast using different flip angle evolution [SPACE]) at 3T.

**Assessment:**

fMRI data at 7T were registered to cortical surfaces reconstructed from 3T structural data acquired in the same subjects. Distortions induced by B_0_ inhomogeneity and gradient nonlinearity (B_0_ and gradient distortions) were evaluated as cortical fallout (misregistration of noncortical areas) and displacement (misregistration along gray matter).

**Statistical Tests:**

Repeated measures analyses of variance with post‐hoc *t*‐tests with Bonferroni correction.

**Results:**

The accuracy of fully corrected fMRI images based on the intensity distribution was 89.2%. Without any corrections, 9.7% of vertices in the whole surfaces were fallout and the average displacement was 0.96 mm for the rest of the vertices. B_0_ and gradient distortion corrections significantly reduced the fallout (to 2.1% and 8.7%) and displacement (to 0.29 mm and 0.86 mm). These corrections were effective even around regions with moderate distortions (the somatosensory and visual cortices for B_0_ distortion, and the anterior frontal, inferior temporal, and posterior occipital cortices for gradient distortion).

**Data Conclusion:**

B_0_ distortion correction is crucial for surface‐based analysis of fine‐resolution fMRI at 7T. Gradient distortion correction should be considered when regions of interest include regions distant from the isocenter of scanners.

**Evidence Level:**

1

**Technical Efficacy Stage:**

1

ULTRA‐HIGH FIELD (UHF) magnetic resonance imaging (MRI) allows us to acquire high‐resolution blood oxygenation‐level dependent (BOLD) functional images by echo‐planar imaging (EPI)^1^ with better contrast^2^, ^3^ and signal‐to‐noise ratio (SNR)^4^, ^5^ compared with conventional field strength (≤3T). UHF also allows imaging with increased spatial resolution, which reduces partial volume effects, thus providing better cortical separation from the surrounding white matter, cerebrospinal fluid (CSF), and pial veins. Spatial specificity of detected brain activity in BOLD functional (f)MRI is also improved at 7T^6^ and it is estimated that the full‐width at half‐maximum of the average point‐spread function is below 1 mm. These advantages have helped in developing cortical column‐^7^ and layer‐based fMRI analyses.^1^, ^8^, ^9^, ^10^ These anatomically informed analyses^11^ depend on a cortical ribbon defined by high‐resolution structural images. Thus, an appropriate correction of BOLD functional image distortions is critical for precise cross‐modal registration.

Because of the higher resonance frequency, UHF‐fMRI is more susceptible to static magnetic field (B_0_) inhomogeneity resulting from differences in magnetic permeability of human brain tissues. Distortion induced by B_0_ inhomogeneity (B_0_ distortion) is seen around the nasal cavity and ear canals^12^, ^13^ in high‐resolution EPI because of the narrow acquisition bandwidth (BW).^14^ Additionally, its appearance is strongly dependent on the phase‐encoding (PE) direction. Conversely, distortion induced by gradient magnetic field nonlinearity (gradient distortion) gradually increases with distance from the isocenter of an MRI scanner. These distortions result in misregistration of EPI to structural images and reduce the accuracy of surface‐based analysis in which the signals in the cortex are selectively sampled and analyzed. In particular, signal sampling at incorrect locations at the surface affects the geodesic distances between points on the cortex. These are neurobiologically more meaningful than simple 3D Euclidean distances, as the cortex is like a complexly folded sheet.^15^ The distortion correction of fMRI images is, therefore, an essential preprocess for surface‐based analysis in UHF‐MRI.

B_0_ inhomogeneity can be estimated and corrected by traversing *k*‐space twice with different PE directions^14^ rather than using the conventional field map method.^12^ This method of correction is available as the tool *topup* in FSL (FMRIB [Functional MRI of the Brain] Software Library, Oxford, UK)^14^, ^16^ (https://fsl.fmrib.ox.ac.uk/fsl/fslwiki/topup). Gradient distortion can be corrected by a voxel displacement map calculated from a gradient coefficient file describing the characteristic of gradient coils as a spherical harmonics expansion.^17^, ^18^ This method of correction is available as the tool *gradunwarp* in FreeSurfer (https://surfer.nmr.mgh.harvard.edu/fswiki/GradUnwarp). Recently, the minimum preprocessing pipelines for the Human Connectome Project (HCP)^19^, ^20^ have incorporated both *topup* and *gradunwarp*, enabling systematic correction for EPI distortions. However, there has been no quantitative analysis of the effects of 7T EPI image distortion corrections on surface‐based analysis in a way that reflects how often noncortical signals are mapped and how far cortical signals are incorrectly mapped onto the surface.

The aim of this study was to quantitatively evaluate the correction of image distortions induced by B_0_ inhomogeneity and gradient nonlinearity on surface‐based analysis in 7T fMRI data.

## Materials and Methods

We first evaluated the accuracy of fully corrected fMRI images based on the intensity distribution of fMRI image voxels within the cortical ribbon defined by the cortical surfaces reconstructed from structural images through HCP Pipelines. In the second step, we quantified the effects as cortical fallout (misregistration of EPI image voxels to the outside of the gray matter in an anatomical image) and cortical displacement (distance between the distorted and corrected EPI image voxels within the surface) by regarding the fully corrected images as ideally corrected ones that corresponded to the structure‐based surfaces and by comparing these surfaces with those replicating fMRI image distortions.

### *Subjects*

This study was approved by the Ethical Committee of the institute and all subjects provided written informed consent. Fifteen healthy individuals aged 19–26 years (eight men and seven women; mean age = 21.9 years; standard deviation [SD] = 1.6 years) participated in the study. None of the subjects had a history of symptoms requiring neurological, psychological, or other medical care.

### *Data Acquisition*

#### *FUNCTIONAL DATA ACQUISITION WITH 7T MRI*

Functional data were acquired with a 32‐channel phased‐array head coil on a Magnetom 7T scanner (Siemens Healthcare, Erlangen, Germany). For whole‐brain coverage at high spatial and typical temporal resolutions, the multiband (MB) gradient‐echo (GRE) EPI sequence^21^ was used in conjunction with the generalized autocalibrating partially parallel acquisition (GRAPPA) technique^22^ with repetition time (TR) = 2000 msec, echo time (TE) = 25 msec, flip angle (FA) = 65°, BW = 1,562 Hz/pixel, in‐plane field of view (FOV) = 192 × 192 mm^2^, matrix = 160 × 160, 128 slices tilted at 15–20° to the transversal plane, 1.2‐mm isotropic voxels, echo spacing (ES) = 0.74 msec, MB acceleration factor = 4, PE undersampling factor GRAPPA = 3, and an anterior‐to‐posterior (AP) PE direction. In this sequence, a single‐band (SB) GRE EPI image was also acquired using a separate SB excitation of the same slices (TR = 2000 msec; volume TR = 8000 msec), with otherwise identical parameters, including PE undersampling factor of 3. This image was used for the evaluations because of its better image quality than MB images and identical distortion to MB images.

Two spin‐echo (SE) EPI datasets with reversed PE directions were also acquired (three volumes with each PE direction) with the same geometric and ES parameters (TR = 11,900 msec, TE = 60 msec, FA = 90°, refocus FA = 180°) as the GRE EPI images. These images, which were referred to as SE field maps in the HCP,^20^ were used to correct B_0_ distortion with FSL's *topup* tool using a method similar to that in Anderson et al^14^ (explained in more detail below).

#### *STRUCTURAL DATA ACQUISITION WITH 3T MRI*

Image intensity is substantially inhomogeneous at 7T due to less uniform excitation (B_1_ inhomogeneity) and larger dielectric effects, especially in the temporal base area. As B_0_ and B_1_ inhomogeneities are less problematic at lower field strength, the structural data for subjects' cortical surface reconstructions were acquired with a 32‐channel phased‐array head coil on a Magnetom Verio 3T scanner (Siemens Healthcare). Two T_1_‐weighted (T_1_w) and two T_2_‐weighted (T_2_w) whole‐brain images were acquired at 0.8‐mm isotropic resolution in each subject to process with HCP Pipelines.^20^ These images were aligned and averaged in each of the T_1_w and T_2_w images to improve the image SNR. T_2_w images were used not only for correcting for bias fields but also for removing veins and dura that were not adequately contrasted with gray matter in T_1_w images. The 3D magnetization prepared rapid acquisition with GRE (MPRAGE) sequence^23^ was used for T_1_w images (TR = 2400 msec, TE = 2.24 msec, inversion time [TI] = 1060 msec, in‐plane FOV = 256 × 240 mm^2^, matrix = 320 × 300, 224 sagittal slices in a single slab, FA = 8°, BW = 210 Hz/pixel, ES = 8.1 msec, PE undersampling factor GRAPPA = 2, and an AP PE), while the 3D sampling perfection with application optimized contrast using different FA evolution (SPACE) sequence^24^ was used for T_2_w images without a fat suppression pulse (same in‐plane FOV, matrix, slices, and PE direction as in the T_1_w images, TR = 3200 msec, TE = 560 msec, BW = 744 Hz/pixel, ES = 3.6 msec, PE undersampling factor GRAPPA = 2, total turbo factor = 334, and echo train length = 1,156). Similarly, two SE field maps with reversed PE directions were also acquired for readout distortion correction with FSL's *FUGUE* tool (TR = 7700 msec, TE = 60 msec, in‐plane FOV = 196 × 196 mm^2^, matrix = 98 × 98, 72 transversal slices, FA = 78°, refocus FA = 160°, BW = 1,822 Hz/pixel, and ES = 0.73 msec).

### *Software*

HCP Pipelines 4.0.0‐alpha.5,^20^ Connectome Workbench 1.2.3 or 1.3.2, FSL 5.0.10, FreeSurfer 5.3.0‐HCP, and HCP *gradunwarp* tool 1.0.3 running on a 64‐bit Linux Operating System (Ubuntu 16.04 LTS or CentOS 6.8 or 6.9) were used for preparation before evaluation of distortion correction such as EPI image distortion correction, registration to structural images, cortical surface reconstruction, and creation of distorted EPI images and distortion‐replicated surfaces. MatLab R2017b (MathWorks, Natick, MA) was used to write home‐made scripts for statistical tests, judgment of cortical fallout of vertices, and measurement of cortical displacements.

### *Preparation for Evaluation of Effects of Distortion Corrections*

#### *CREATION OF FULLY CORRECTED ECHO‐PLANAR IMAGES*

**FIGURE 1:**
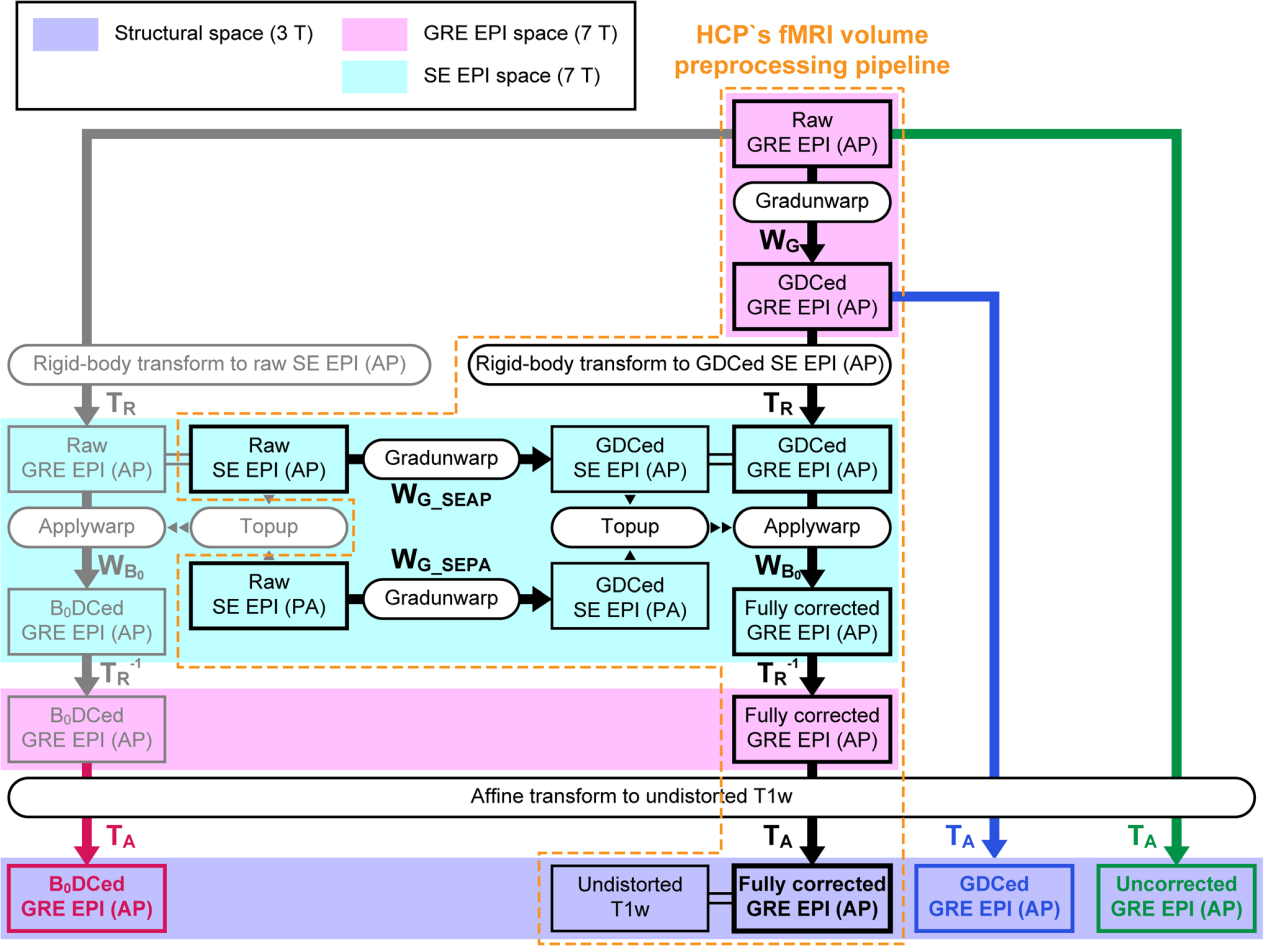
Flow chart of the Human Connectome Project (HCP) pipeline and its modification. A zone surrounded by an orange dotted line indicates the process of the HCP's functional MRI (fMRI) volume preprocessing pipeline. The pipeline fully corrects the distortion of the gradient‐echo (GRE) echo‐planar imaging (EPI) images with the anterior‐to‐posterior (AP) phase‐encoding direction to register to an undistorted T_1_‐weighted (T_1_w) structural image. Boxes indicate output images by a process, and rounded boxes indicate script names. Black arrows indicate the application of the warp field (*W*) or transform matrix (rigid‐body transform; *T*_*R*_ or affine transform; *T*_*A*_) to images. *W* with subscripts *G* and *B*_*0*_ shows that the warp field is for gradient distortion correction (GDC) and B_0_ distortion correction (B_0_DC), respectively. *W* with subscripts *G_SEAP* and *G_SEPA* indicates that the warp field is applied to correct raw spin‐echo (SE) images acquired with AP and PA (posterior‐to‐anterior) phase‐encoding directions for gradient distortion, respectively. Transform matrices with the superscript –*1* indicate that the matrices are inverse ones. Purple, magenta, and aqua zones denote 3T structural, 7T GRE EPI, and 7T SE EPI spaces. Different “*T*_*A*_”s were generated for registration of fully corrected GRE EPI (black), raw GRE EPI (green), gradient distortion‐corrected (GDCed) EPI (blue), and B_0_ distortion‐corrected (B_0_DCed) GRE EPI (red).

We created fully corrected EPI images through HCP's fMRI volume preprocessing pipeline^20^ (zone surrounded with an orange dotted line in Fig. 1). A warp field to correct gradient distortion of an SB GRE EPI image was first obtained using HCP *gradunwarp* tool (black *W*_*G*_ in Fig. 1), which was part of FreeSurfer. This calculated the amount of gradient distortion based on a scanner‐specific gradient coefficient file.^18^ *Gradunwarp* was also applied to the two SE EPI images with reversed PE directions (*W*_*G_SEAP*_ and *W*_*G_SEPA*_ in Fig. 1). The amount of B_0_ distortion was next estimated from these gradient distortion‐corrected SE EPI images using FSL's *topup* tool^14^ (black *W*_*B0*_ in Fig. 1). The resulting warp fields were then sequentially applied to a raw SB GRE EPI image to fully correct the image with the intensity correction using Jacobian modulation.^14^ Rigid‐body transformation was used to remove a misalignment between SE and GRE EPI images due to subject's head motion in discrete runs (black *T*_*R*_ in Fig. 1). For registration of the corrected SB GRE EPI image to a T_1_w one (black *T*_*A*_ in Fig. 1), a transform matrix was calculated by sequentially using two registration tools: FSL's *BBR* and FreeSurfer's *BBRegister*.^25^ Instead of rigid‐body transform, affine transform was used for the registration to minimize interscanner gradient distortion correction (GDC) errors,^26^ as we used 3T and 7T scanners for structural and EPI acquisitions, respectively.

#### *CREATION OF DISTORTED ECHO‐PLANAR IMAGES*

For the evaluation of distortion corrections, we prepared three types of distorted SB GRE EPI images for each subject: uncorrected, gradient distortion‐corrected, and B_0_ distortion‐corrected. A raw SB GRE EPI image was directly used as an uncorrected EPI image (green bold arrow in Fig. 1). For a gradient distortion‐corrected image, a corrected image just after applying *gradunwarp* to a raw image was utilized (blue bold arrow in Fig. 1). A B_0_ distortion‐corrected image was created by skipping GDC and applying only a warp field output by *topup* to a raw image (gray and red bold arrows in Fig. 1). The intensity of these partially distortion‐corrected images was corrected in a similar way to the fully corrected images described above. A transform matrix that the fMRI volume preprocessing pipeline normally outputs to register a fully corrected (undistorted) SB GRE EPI image was not used (black *T*_*A*_ in Fig. 1) for registrations of these distorted images to the structural data because the estimated transform matrix (black *T*_*A*_) is influenced by the preceding distortion correction procedures. An affine transform matrix calculated for each distorted image (uncorrected, gradient distortion‐corrected, and B_0_ distortion‐corrected) was employed for conversion from distorted EPI image space to structural image space (green, blue, and red “*T*_*A*_”s in Fig. 1, respectively).

#### *CREATION OF DISTORTION‐REPLICATED CORTICAL SURFACES AND RIBBONS*

**FIGURE 2:**
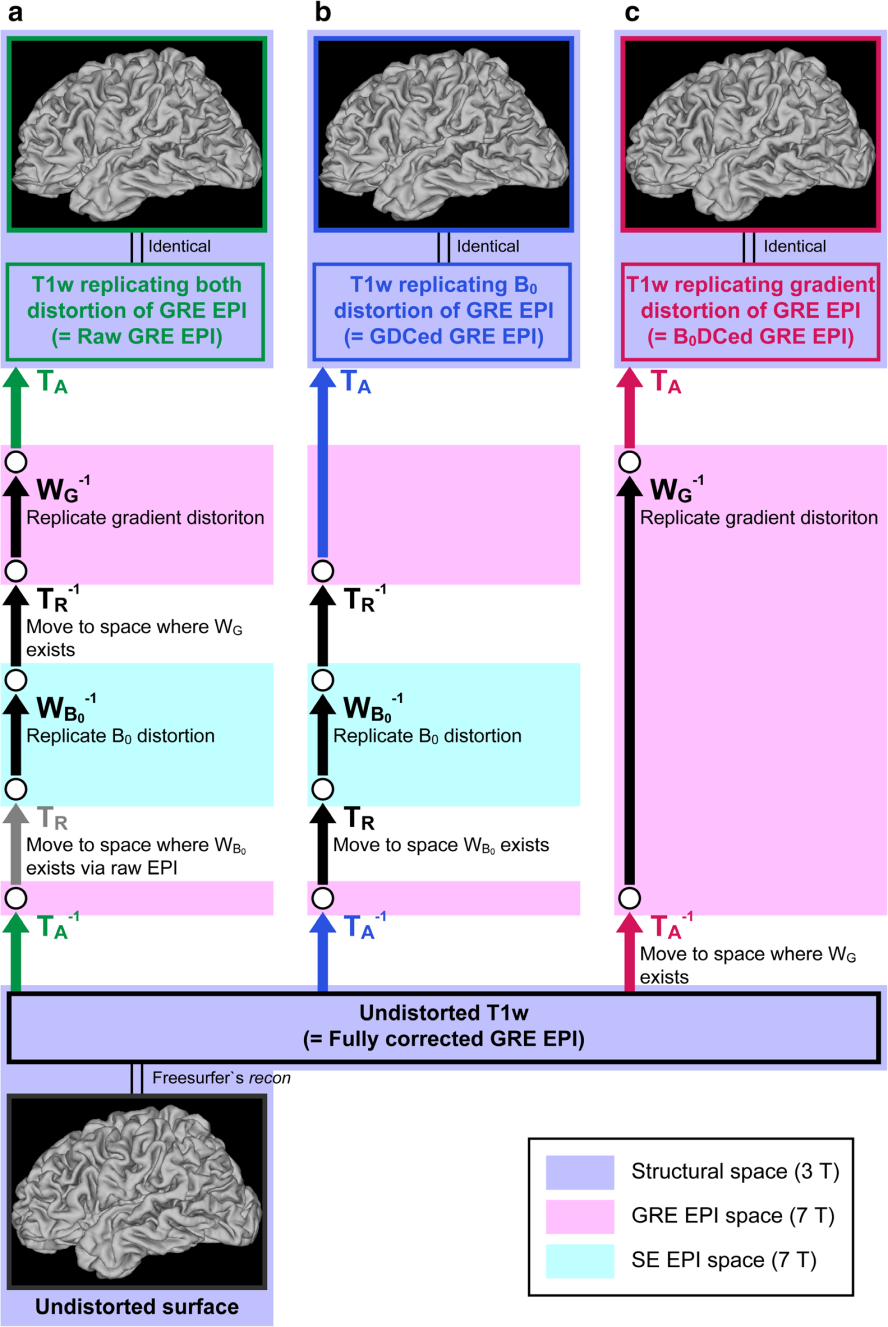
Flow chart of echo‐planar imaging (EPI) image distortion replication. The distortion correction processes shown in Fig. 1 were reversely applied to the undistorted T_1_‐weighted (T_1_w) image to create cortical surfaces. (**a**) Both B_0_ distortion and gradient distortion, (**b**) B_0_ distortion only, and (**c**) gradient distortion only are replicated. Abbreviations are the same as in Figure 1.

Undistorted native cortical surfaces were created from the 3T structural images through a series of the original HCP's surface reconstruction pipelines,^20^ where gradient and readout distortions were corrected. Meanwhile, surfaces where EPI image distortions were replicated for each distortion condition in each subject were generated as follows. To distort the undistorted native surface, inverse warp fields, upsampled to the same resolution as the structural images (0.8 mm isotropic resolution), were first made from the warp fields for distortion corrections. A warp field for creating B_0_ distortion‐replicated (gradient distortion‐corrected) surfaces was generated by concatenating the inverse warp field to replicate B_0_ distortion, inverse affine and rigid‐body transform matrices to convert from the structural image space to the SE EPI space where the inverse warp field existed, and rigid‐body and inverse affine transform matrices to convert back to the structural image space (Fig. 2b). Then, by applying this new warp field to the undistorted native surfaces with the command *surface‐apply‐field* of the Connectome Workbench, surfaces replicating the B_0_ distortion of the EPI images were created. Similarly, a warp field for creating gradient distortion‐replicated (B_0_ distortion‐corrected) surfaces was generated by concatenating the inverse warp field to replicate gradient distortion, inverse affine transform matrix to convert from the structural image space to the GRE EPI space where the inverse warp field existed, and the affine transform matrix to convert back to the structural image space (Fig. 2c). Moreover, for a warp field creating surfaces where distortions of uncorrected EPI images were replicated, all of these transform matrices and inverse warp fields were concatenated (Fig. 2a). Identical procedures were applied to the undistorted cortical ribbons to generate distortion‐replicated cortical ribbons.

### *Evaluation of Distortion Correction Effects*

#### *CALCULATION OF ECHO‐PLANAR IMAGE DISTORTION*

The absolute amounts of B_0_ and gradient distortions of the EPI images were calculated from the x, y, and z components of the warp field output by *topup* for B_0_ distortion correction (B_0_DC) and *gradunwarp* for GDC in each subject, respectively. The affine transform matrix for the registration of the fully corrected SB EPI image to the T_1_w image was applied to these calculated data to overlay onto the T_1_w image. The rigid‐body transform matrix to convert the SE EPI space to the GRE EPI space was also applied to the calculated data for the B_0_ distortion, as the warp field for the calculation was in the SE EPI space.

#### *EVALUATION OF ACCURACY OF FULLY CORRECTED ECHO‐PLANAR IMAGES*

The overall efficiency of the full distortion correction was evaluated before the surface‐based evaluations. Fully corrected EPI images were regarded as perfectly correct (corresponding to the structural images) and were thus used as a reference against other partially corrected and uncorrected ones in the surface‐based evaluation.

Voxels in the cortical ribbon defined by the structural images were first extracted from the fully corrected SB GRE EPI image in each subject to generate a normalized intensity histogram of these voxels as a probability density. Given that all these voxels are of the gray matter, the shape of the histogram should have a single peak with a normal probability density function. However, this may not be the case because of white matter and CSF contamination resulting from imperfect distortion correction, and intensity dropout, which is inevitable in EPI images. Moreover, the partial volume effect may broaden the bottom of the single peak. Therefore, considering these factors, we fitted the probability density function with four normal distribution functions for the four components: gray and white matter, CSF, and intensity dropout using MatLab's *mle* function. Maximum likelihood estimation was performed to estimate 12 parameters: weights, means, and SDs for these four normal distribution functions. An estimated intensity histogram *F(x)* as probability density for normalized intensity *x* was calculated according to the following equations:

$$F\left(x\right)=w_{g}f\left(x,\mu_{g},\sigma_{g}\right)+w_{w}f\left(x,\mu_{w},\sigma_{w}\right)+w_{f}f\left(x,\mu_{f},\sigma_{f}\right)+w_{d}f\left(x,\mu_{d},\sigma_{d}\right)$$

$$w_{g}+w_{w}+w_{f}+w_{d}=1$$

where *f(x*, *μ*, *σ)* is a normal distribution function for the average *μ* and SD *σ*, *μ*_*g*_, *μ*_*w*_, *μ*_*f*_, *μ*_*d*_, *σ*_*g*_, *σ*_*w*_, *σ*_*f*_, and *σ*_*d*_ are intensity averages and SDs of the gray and white matter, CSF, and dropout, respectively, and *w*_*g*_, *w*_*w*_, *w*_*f*_, and *w*_*d*_ are weights for the same four components.

To confirm that the fitting was valid, simulated spatial distribution maps for the four components were created for each subject. Voxels in the cortical ribbon were classified into four categories based on the frequency ratio of the four components in each bin (0.1 SD). For example, when a bin is composed of 1000 voxels and the frequency ratio of the gray matter is 0.8, 800 voxels of these voxels were randomly labeled as gray matter.

#### *CALCULATION OF CORTICAL FALLOUT AND CORTICAL AND EUCLIDEAN DISPLACEMENTS*

A Euclidean displacement map was obtained by calculating spatial distances between corresponding vertices of undistorted and distorted surfaces and mapping them onto the undistorted surfaces for each distortion condition in each subject.

**FIGURE 3:**
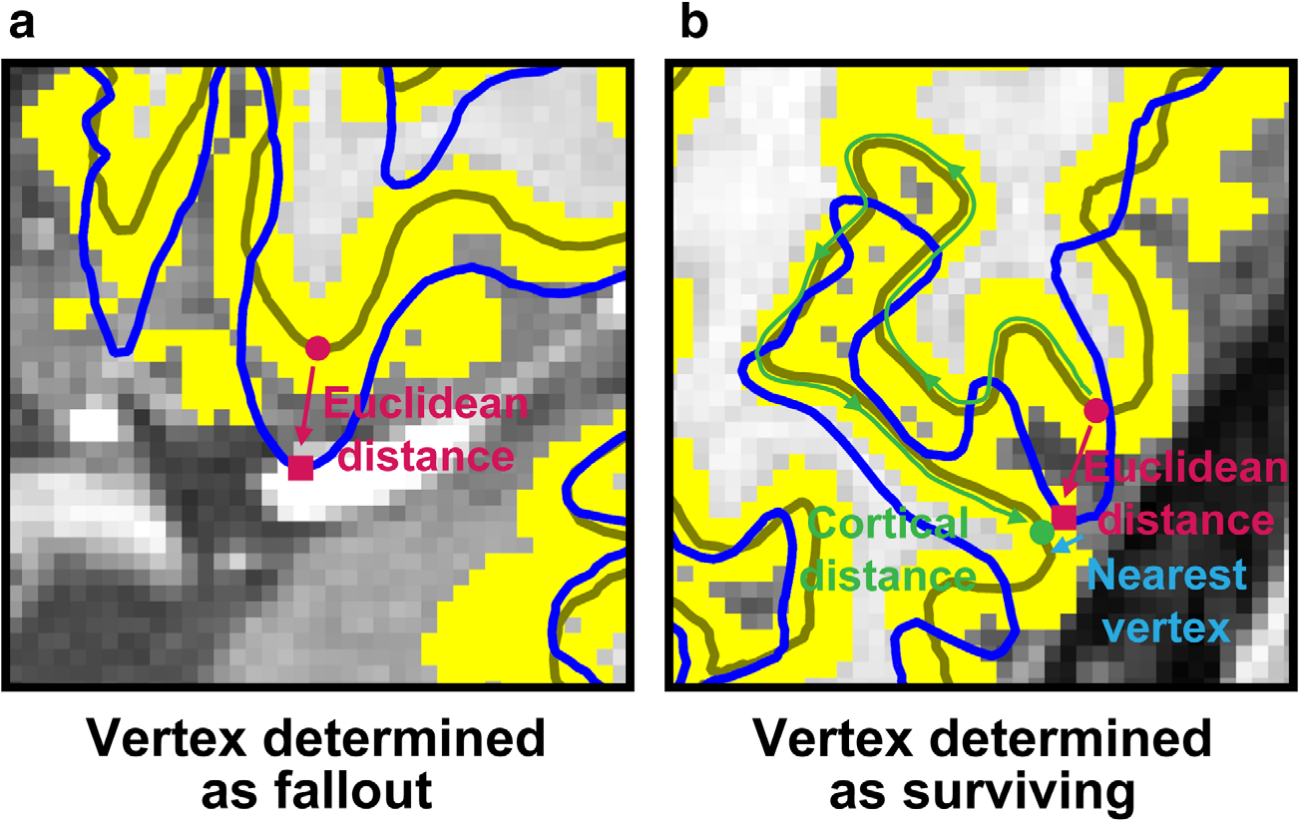
Examples of a fallout (**a**) and surviving vertices (**b**). The trace lines of an original undistorted mid‐thickness surface and distorted one are shown as olive and blue lines, respectively. A cortical ribbon, a gray matter zone positioning between the undistorted white and pial surfaces (these trace lines are not shown), is denoted as yellow voxels. A target vertex on the undistorted mid‐thickness surface (magenta filled circle on the olive line) moves onto the distorted mid‐thickness surface (magenta filled square on the blue line), which is outside the cortical ribbon in (a), thus determined as a fallout. Meanwhile, in (b), the correspondent vertex is inside the ribbon. In this case, the nearest vertex on the undistorted mid‐thickness surface to the correspondent vertex is searched (green filled circle). A cortical distance between the target and searched vertices is measured on the undistorted mid‐thickness surface (green curved arrowed line along the olive line), while the Euclidean distance between them is shown with a magenta arrowed line.

We needed to determine whether each vertex of the distorted “midthickness” surface, which was the intermediate surface between “white” and “pial” surfaces of FreeSurfer, was located in or out of the undistorted cortical ribbon for each distortion condition in each subject. Therefore, x, y, and z coordinate values of the vertex were first obtained in the native volume space after the “acpc alignment” step.^20^ The vertex was classified as a fallout, when the ribbon did not contain the vertex (Fig. 3a). Meanwhile, for the vertex contained in the ribbon (surviving), we searched the vertex nearest to the coordinate values on the distorted surface and the vertex correspondent to the nearest vertex on the undistorted surface (Fig. 3b). A cortical distance between these vertices was measured and mapped onto the undistorted surface as a cortical displacement. A cortical fallout map was finally obtained by assigning “1”s to fallout vertices on the undistorted surface.

Surface maps of the cortical fallout and cortical and Euclidean displacements for each distortion condition from the 15 subjects were transformed to the 164 k‐CIFTI (Connectivity Informatics Technology Initiative) format^20^ with the application of MSMSulc^26^ and averaged over the subjects in each vertex to obtain group maps. Group‐averaged maps of the cortical thickness and curvature, which expressed whether the surface was convex or concave, were also calculated with the output data from the HCP structural pipelines. The displacement ratio was defined as the ratio between the cortical and Euclidean displacements of each vertex. The group displacement ratio map was created by computing the group cortical displacement map divided by the group Euclidean map in each distortion condition.

The fallout rate was calculated by dividing the number of fallout vertices by that of the valid ones in the 164 k CIFTI format in each subject and then averaging across subjects in each distortion condition to calculate the group fallout rate. Additionally, the average cortical displacement of each subject was obtained by averaging the values stored in all vertices, except fallout ones, of the surface map in each condition.

### *Statistical Tests*

We applied repeated measures analysis of variance (ANOVA) to each of the 15 subjects' cortical fallout rates and displacements among the three distortion conditions. A Bonferroni correction was applied to correct for multiple comparisons; *α* was set at 0.0167 for these conditions in post‐hoc paired *t*‐tests.

## Results

### *Echo‐Planar Image Distortions*

**FIGURE 4:**
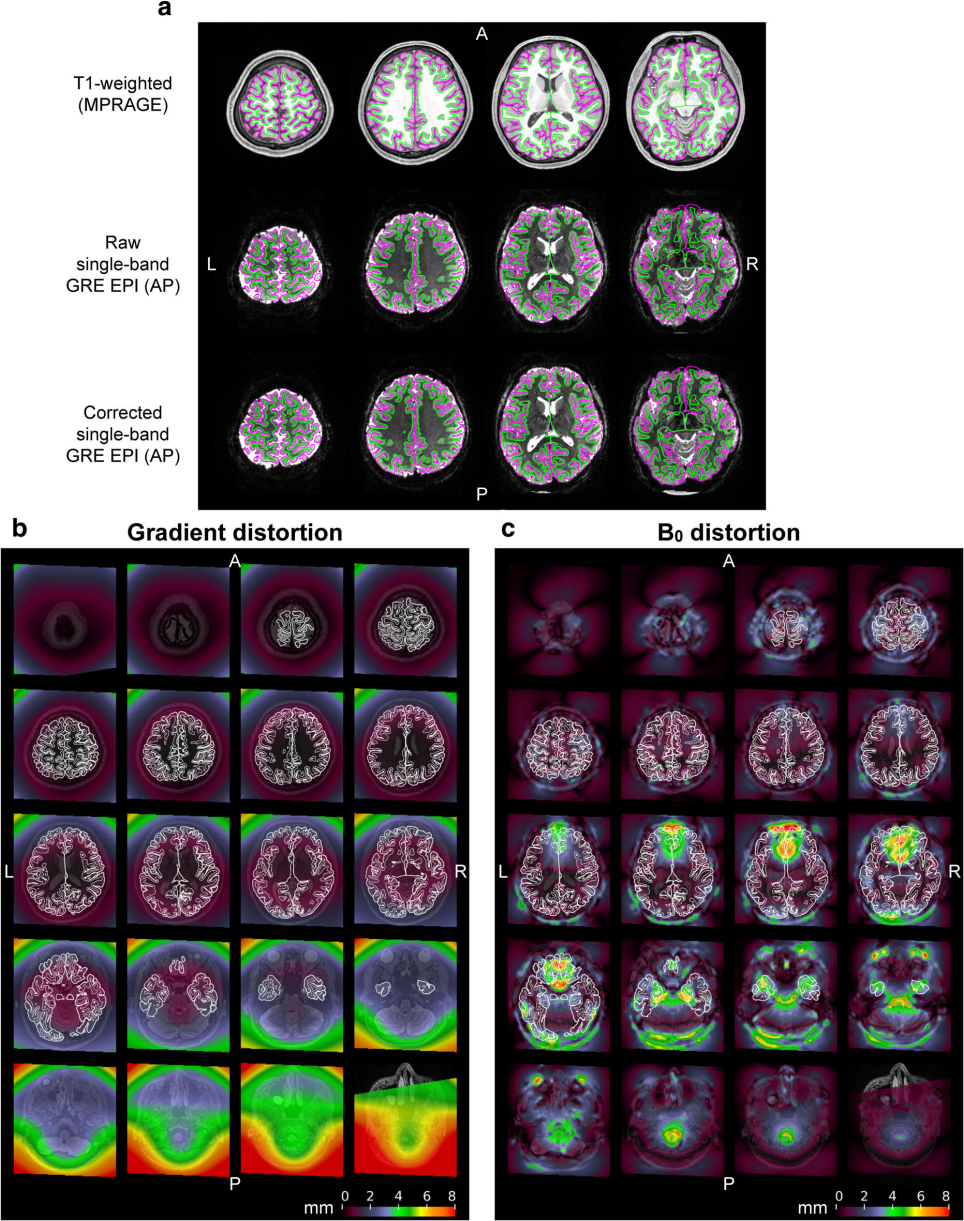
Distortions of echo‐planar images. (**a**) Raw and corrected echo‐planar images are compared with a structural image at four slice positions in a subject. A T_1_‐weighted structural image acquired at 3T is shown in the first row. In the second row, a raw single‐band (SB) gradient‐echo (GRE) EPI image acquired with the anterior‐to‐posterior (AP) phase‐encoding direction at 7T is registered onto the structural image (first row). An echo‐planar image in which distortion corrections are applied to the raw one (second row) is registered in the third row. Green and magenta lines denote white and pial surfaces created with FreeSurfer. The amount of distortions induced by gradient nonlinearity and B_0_ inhomogeneity are shown in (**b,c**), respectively. Colors on these maps indicate how much distortion each position in the brain experiences in the raw echo‐planar image in mm. Thin and thick white lines denote the same white and pial surfaces, respectively.

Figure 4a shows a comparison of a T_1_w image (top row) with a raw SB GRE EPI image acquired with the AP PE direction (middle row) in four different slices in a single subject. A distortion‐corrected EPI image with *topup* is also shown in the bottom row. Mismatches between the gray matter region of the EPI image and trace lines of gray matter defined by the structural images were observed, particularly, around the frontal and occipital poles, and the orbito‐frontal cortex (OFC). The amount of distortions experienced at each position because of gradient nonlinearity and B_0_ inhomogeneity are shown in Fig. 4b,c, respectively. Gradient nonlinearity induced distortions over 2 mm were seen around the frontal and inferior occipital regions, although the distortions were milder than B_0_ inhomogeneity‐induced ones. In addition, the inferoposterior part of the cerebellum was distorted by ~4 mm. Meanwhile, the heaviest B_0_ distortions were observed around the OFC near the nasal cavity and the inferior temporal cortex (ITC) near the ear canal (~10 mm). Distortions over 5 mm were also seen around the occipital pole, and several‐millimeter distortions occurred in many other parts.

### *Accuracy of Fully Corrected Echo‐Planar Images*

**FIGURE 5:**
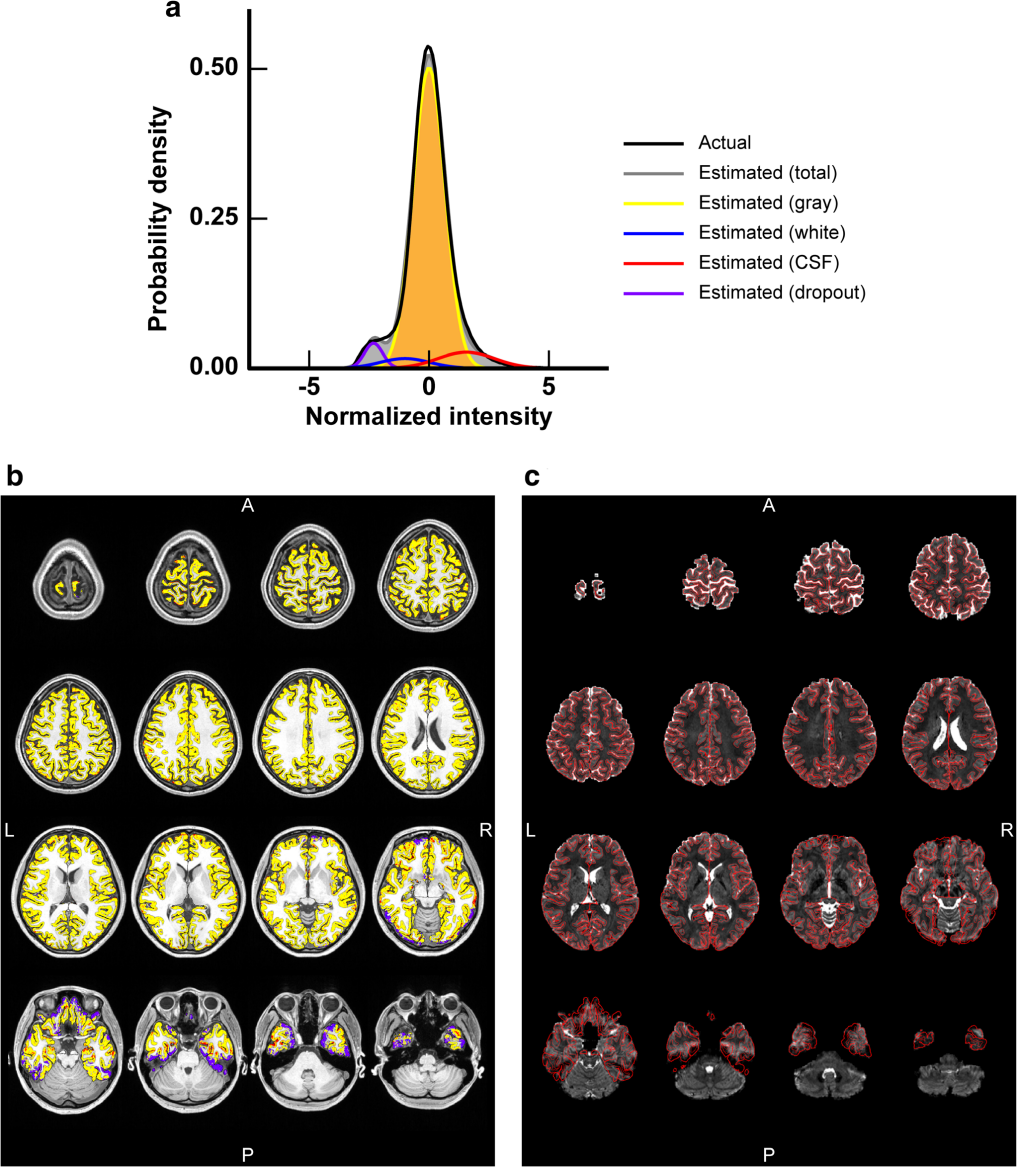
Evaluation of distortion correction accuracy of a distortion‐corrected gradient‐echo (GRE) echo‐planer image in a typical subject. (**a**) A normalized intensity histogram of voxels of a single‐band (SB) GRE echo‐planer image (**c**) that locate within a cortical ribbon defined by structural images is shown as a probability density. The black curve is an actual distribution of the voxel intensity, while the gray one is the total estimated distribution by summing estimated distributions of gray and white matter, cerebrospinal fluid (CSF), and intensity dropout (denoted by yellow, blue, red, and purple curves, respectively). (**b**) The spatial distribution of voxels that are categorized into the four types (gray and white matter, CSF, and intensity dropout) based on fitting to the normalized intensity histogram is shown on a T_1_‐weighted structural image. Voxel colors are the same as in (a). (c) The SB GRE EPI image used for the estimation is shown with borders of the cortical ribbon (red lines).

The black curve in Fig. 5a indicates the normalized intensity histogram of voxels in the cortical ribbon defined by the structural images as a probability density in a typical subject. Estimated histograms for the four components are also shown with yellow, blue, red, and purple curves, respectively. The simulated distribution of these four components in the ribbon is shown in Fig. 5b. Yellow voxels are prominently distributed in the ribbon with a scattering of blue and red voxels, consistent with the notion that the yellow voxels correspond to gray matter (see also Fig. 5c). As the peak density of the blue component was lower than that of the yellow component, which was in turn lower than the peak of the red component, the blue and red voxels corresponded to white matter and CSF, respectively. A substantial bump seen in the left side of the histogram (purple curve in Fig. 5a) was distributed around the frontal pole, OFC, and ITC where intensity dropout was prominent (Fig. 5c). Overall, these data indicate that the estimated components of the probability density represent the corresponding tissues of gray and white matter, CSF, and intensity dropout.

Table 1 shows the distortion correction accuracy for fully corrected EPI images in all subjects. As the average *w*_*d*_ was 0.048, 4.8% of the voxels in the cortical ribbon lost signal in the fully corrected EPI images. The simulation (Fig. 5) showed that voxels labeled as white matter and CSF were diffusely distributed in the ribbon and occupied 1.9% (average *w*_*w*_ = 0.019) and 8.3% (average *w*_*f*_ = 0.083) of its volume, respectively. Altogether, the average distortion correction accuracy was 89.2 ± 2.0%.

**TABLE 1:** Distortion Correction Accuracy and Weights of Gray Matter, White Matter, Cerebrospinal Fluid (CSF), and Intensity Dropout (*w*_*g*_, *w*_*w*_, *w*_*f*_, and *w*_*d*_, respectively) for Fully Corrected Echo‐Planar Images in 15 Subjects

Subject number	Gray (*w*_*g*_)	White (*w*_*w*_)	CSF (*w*_*f*_)	Dropout (*w*_*d*_)	Accuracy (%)
01	0.840	0.049	0.061	0.050	88.4
02	0.887	0.011	0.054	0.048	93.1
03	0.872	0.022	0.074	0.032	90.1
04	0.862	0.010	0.084	0.044	90.1
05	0.827	0.055	0.081	0.037	85.9
06	0.836	0.043	0.080	0.041	87.2
07	0.831	0.074	0.069	0.027	85.4
08	0.846	0.000	0.098	0.057	89.7
09	0.857	0.000	0.100	0.043	89.5
10	0.848	0.017	0.091	0.044	88.7
11	0.838	0.005	0.099	0.058	88.9
12	0.842	0.000	0.100	0.058	89.4
13	0.860	0.000	0.080	0.060	91.4
14	0.855	0.002	0.077	0.067	91.5
15	0.843	0.000	0.100	0.057	89.4
Average	0.850	0.019	0.083	0.048	89.2
Standard deviation	0.016	0.024	0.015	0.011	2.0

### *Group Average Effects of Distortion Corrections on Surface‐Based Analysis*

**FIGURE 6:**
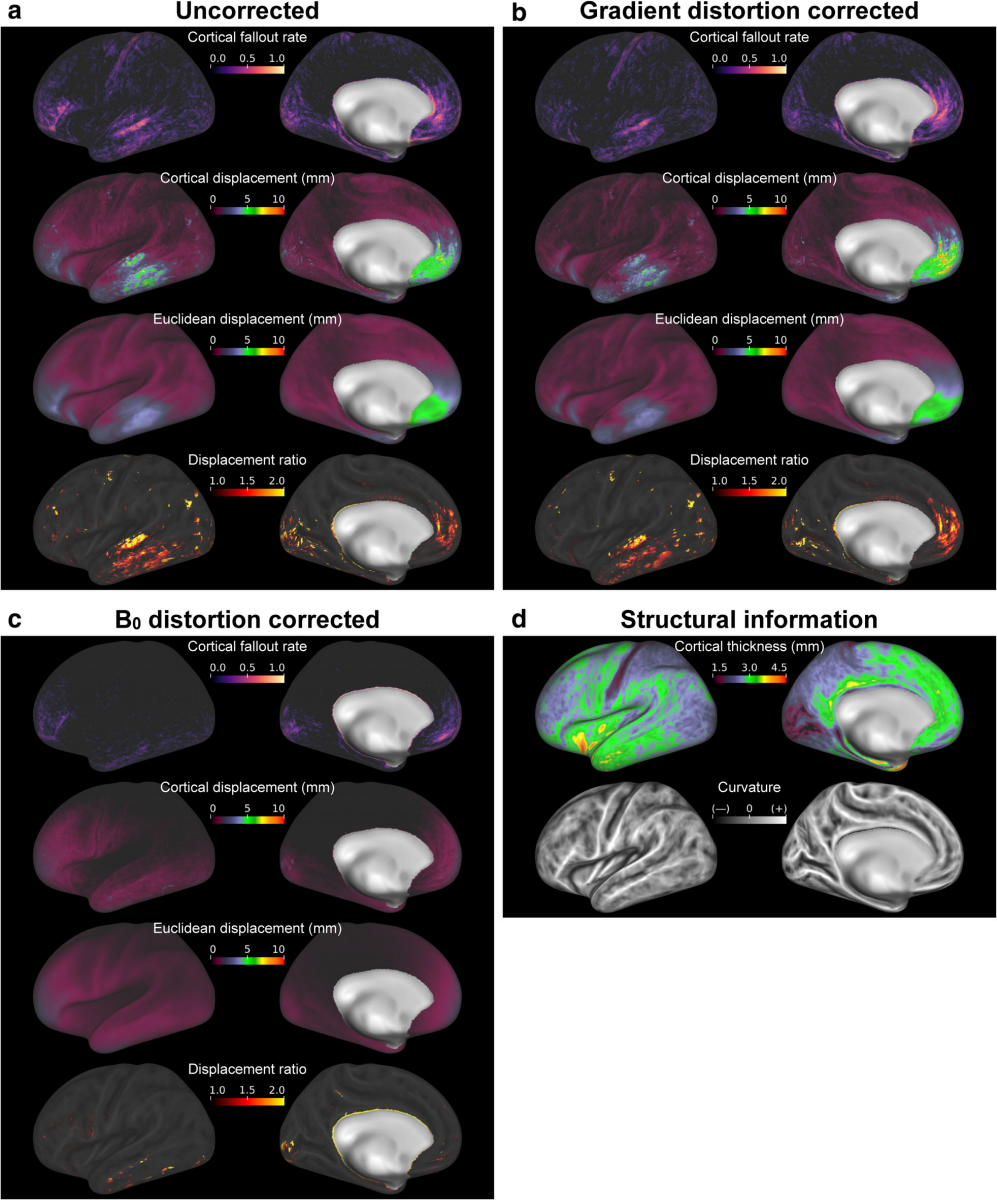
Group‐averaged effects of distortion corrections on the cortical surface‐based analysis. Panels (**a,b,c**) correspond to the effects in case that echo‐planar images acquired with the anterior‐to‐posterior phase‐encoding direction were registered to the structural image, with no correction, gradient distortion correction (GDC), and B_0_ distortion correction (B_0_DC), respectively. Maps of the cortical fallout rate, cortical displacement, Euclidean displacement, and displacement ratio are shown in the first to fourth rows of each panel. The fallout rate maps indicate how frequently each vertex was categorized as data from extracortical voxels. Additionally, maps of cortical thickness and curvature averaged over 15 subjects are shown in (**d**).

**FIGURE 7:**
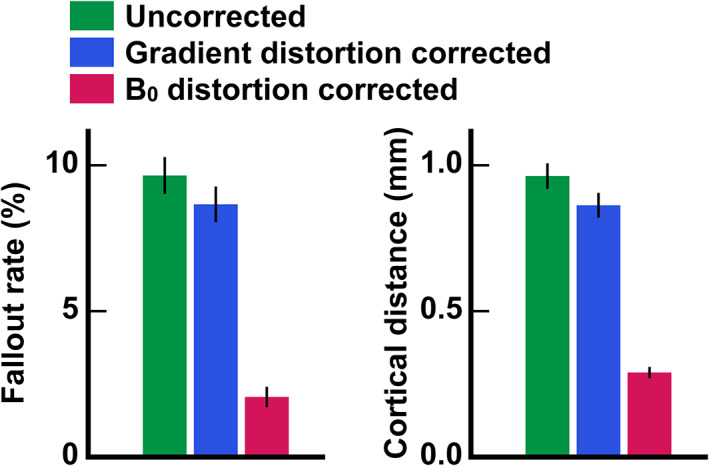
Group‐averaged cortical fallout rates and displacements across vertices in three distortion conditions. Post‐hoc paired *t*‐tests for 15 subjects indicated significant differences in both the cortical fallout rate and displacement among the conditions. Error bars indicate standard errors of the mean across subjects.

Figure 6a shows the effects of uncorrected distortion on surface‐based analysis when the uncorrected SB GRE EPI images were registered to the structural images. Cortical fallout was obvious in the OFC, ITC, occipital cortex, and posterior bank of the central sulcus. In the former two cortices, cortical and Euclidean displacements and their ratio were large, while, in the latter two regions, the ratio was large in spots despite the fact that these displacements were small. The group‐averaged cortical fallout rate and displacement were 9.7 ± 2.5% and 0.96 ± 0.17 mm in the whole cortex, respectively (see also green bars in Fig. 7).

Figure 6b shows the effects of GDC. The cortical fallout was again obvious in the OFC, ITC, occipital cortex, and posterior bank of the central sulcus. The maps of Euclidean and cortical displacements and the displacement ratio were also similar to those in Fig. 6a. Cortical displacement was typically smaller than that in Fig. 6a, except in part of the OFC. Reflecting these results, the group‐averaged cortical fallout rate and displacement were significantly smaller (8.7 ± 2.4% and 0.86 ± 0.16 mm, respectively; see also blue bars in Fig. 7) than those in the uncorrected condition.

Finally, the effects of B_0_DC are shown in Fig. 6c. Fallout was seen in the anterior frontal cortex, ITC, and posterior occipital cortex (POC). Cortical and Euclidian displacements were relatively large in these regions, and the distance ratio was large in spots in the ITC and POC. The group‐averaged cortical fallout rate and displacement was the lowest in this case (2.1 ± 0.7% and 0.29 ± 0.07 mm, respectively; see also magenta bars in Fig. 7).

Figure 7a shows the comparisons of group‐averaged cortical fallout rates for the three distortion conditions. The repeated measures ANOVA for 15 subjects indicated statistically significant differences among the three conditions (*F*(2,28) = 133.95, *P* < 0.05). The post‐hoc paired *t*‐tests revealed significant differences among all pairs of conditions (*P* < 0.0167 with Bonferroni correction, *t*(14) = 5.45 between the uncorrected and GDC conditions, *t*(14) = 12.35 between the uncorrected and B_0_DC conditions, and *t*(14) = 11.13 between GDC and B_0_DC conditions). The comparisons of group‐averaged cortical displacements among the three distortion conditions are shown in Fig. 7b. Again, the repeated measures ANOVA indicated statistically significant differences among the conditions (*F*(2,28) = 241.71, *P* < 0.05). The post‐hoc paired *t*‐tests revealed significant differences among all pairs of conditions (*P* < 0.0167 with Bonferroni correction, *t*(14)= 9.13 between the uncorrected and GDC conditions, *t*(14) = 17.03 between the uncorrected and B_0_DC conditions, and *t*(14) = 14.36 between GDC and B_0_DC conditions).

### *Individual Effects of Distortion Correction on Surface‐Based Analysis and Volume Data*

**FIGURE 8:**
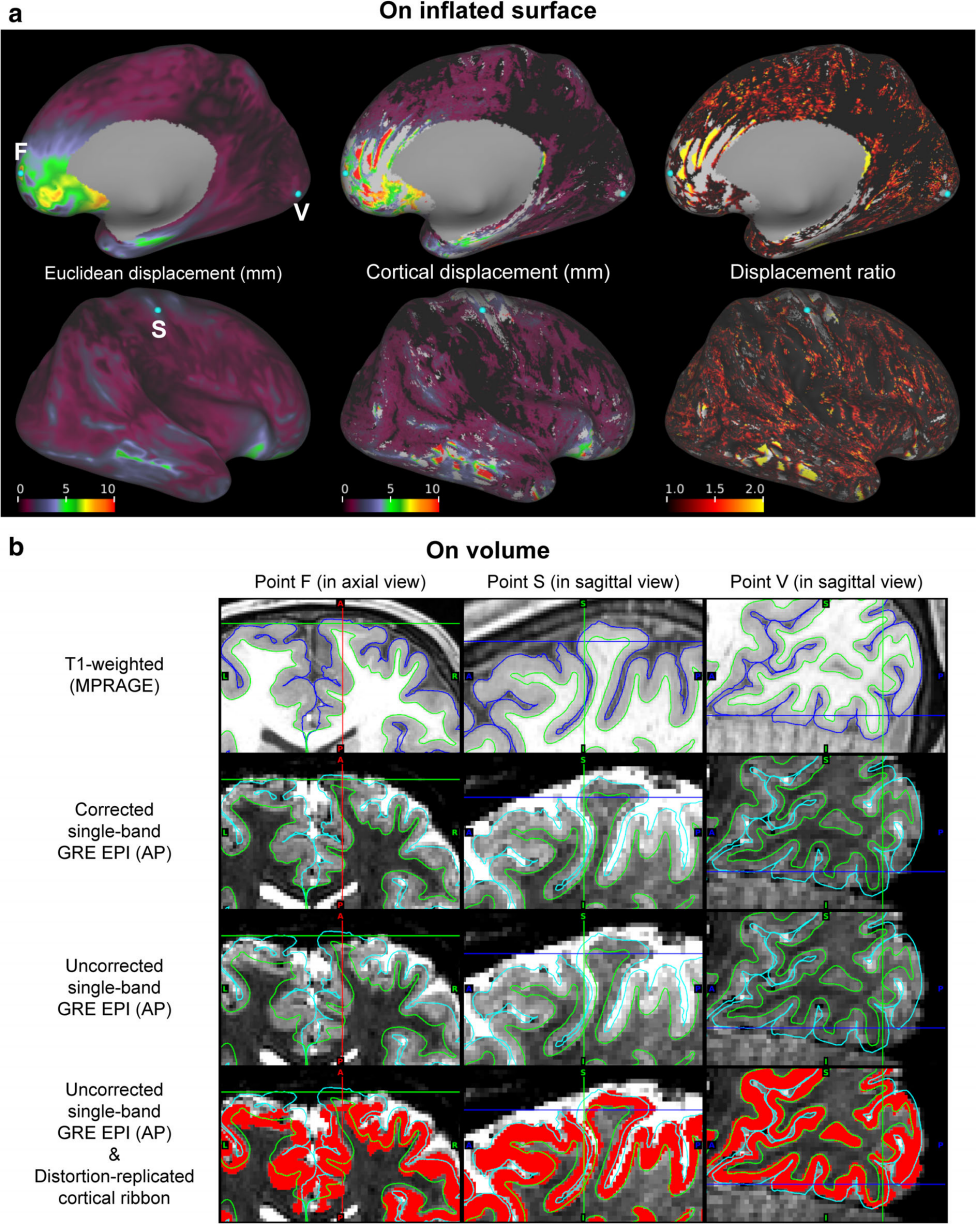
Individual effects of distortion corrections on cortical surface‐based analysis. (**a**) Maps of Euclidean (left column) and cortical displacements (middle column), and displacement ratio (right column) are shown on the right inflated surface of a typical subject. These maps from medial (top row) and lateral views (bottom row) are aligned. (**b**) Cross‐sectional views of the points F, S, and V in (a) are shown in the left, center, and right columns, respectively. Crosshairs correspond to these points. In each cross‐sectional view, T_1_‐weighted and corrected single‐band (SB) gradient‐echo (GRE) echo‐planar images are shown in the first and second rows, respectively. The uncorrected versions of the echo‐planar images are also shown in the third row. The distortion‐replicated cortical ribbon denoted in red is overlaid on the uncorrected images in the fourth row. Green and blue or aqua lines indicate pial and white surfaces created with FreeSurfer, respectively.

Figure 8a demonstrates maps of Euclidean and cortical displacements and their ratio in the right hemisphere of a typical subject when no distortion corrections were applied to the EPI image. Euclidean and cortical displacements were large in the medial frontal cortex (MFC), OFC, and ITC. Cortical fallouts were seen not only around these cortices but also around the central sulcus and part of the occipital cortex.

The cortical fallouts around the AFC, posterior bank of the central sulcus, and POC (left, middle, and right columns, respectively) are reflected in the volume data from the same typical subject in Fig. 8b. In the T_1_w images in the first row, gray matter voxels are located between two trace lines: pial and white surfaces. When these lines are overlaid on the corrected SB EPI images, we find gray matter voxels included between them (see images in the second row). In contrast, voxels from white matter or CSF intruded into the gray matter zones in the uncorrected SB EPI image registered to the structural image (see images in the third row). This can be more clearly seen from images in the fourth row, where distortion‐replicated cortical ribbon is overlaid on the distorted EPI images and the correct trace lines. The point F in the AFC includes signals outside the CSF (quite low voxel intensity) because of a distortion to the posterior (see the left column). We also found a cortical displacement over 5 mm in the medial area. Meanwhile, the points S and V in the somatosensory and visual cortices included signals from the CSF and white matter (higher and lower voxel intensity than gray matter), as shown in the middle and right columns, respectively.

## Discussion

This study quantified the effects of geometric distortion corrections on surface‐based analysis in terms of cortical fallouts and displacements, instead of simple 3D Euclidean displacements. To this end, cortical surfaces replicating distorted EPI images were compared with those reconstructed from undistorted structural images, which were regarded as surfaces replicating fully corrected EPI images, that is, ideally corrected ones. However, since the accuracy of fully corrected EPI images was unknown, it was first evaluated based on intensity distributions of voxels within a cortical ribbon. The simulated spatial distribution map obtained by fitting the probability density function with four normal distribution functions for gray and white matter, CSF, and intensity dropout exhibited clear intensity dropout around the OFC and ITC, as expected. Some voxels labeled as white matter and CSF were randomly scattered in gray matter, while most were located on the edge. SB GRE EPI images, the least noisy EPI images, are therefore imperfect for voxel classification of brain tissues. Thus, the actual distortion correction accuracy for fully corrected EPI images may be greater than the 89.2% estimated.

Cortical fallout and displacement have allowed us to clearly delineate the impact of subtle distortions. In the comparison between undistorted and distortion‐replicated cortical surfaces, the most striking finding was that even moderate distortions can yield important cortical fallout and displacement. Hitherto, for distortion correction evaluations, distorted EPI images were simply compared to structural or corrected EPI images, or the amounts of distortions were shown or overlaid onto structural images.^13^, ^20^, ^27^, ^28^, ^29^ As with the other approaches, B_0_ distortion was indirectly evaluated by presenting effects on functional connectivity,^30^ and the effect of gradient distortion correction was assessed by evaluating voxel‐based image intensity reproducibility.^18^ These studies remind us of the importance of distortion correction in brain regions where distortions are prominent, such as the OFC and ITC for B_0_ distortion. Our results supported these findings, showing a high cortical fallout rate and large cortical displacement around these regions. Additionally, prominent cortical fallouts were observed in the posterior bank of the central sulcus (the primary somatosensory cortex) and visual cortex, where distortions were supposed to be mild or moderate without B_0_DC, unlike areas near the nasal cavity and ear canals.^12^, ^13^ The primary somatosensory cortex is one of the thinnest cortices.^31^, ^32^ The visual cortex is also thin,^33^, ^34^ with complex gyri and sulci running throughout.^35^ Due to these structural factors, small distortions in the AP direction resulting from PE can easily cause a fallout.

Across the whole brain, we observed far higher cortical fallout rate and larger cortical displacement with only GDC compared to only B_0_DC, indicating that B_0_ distortion was a major cause of distortions. The effect of GDC in our study was small. Glasser et al^20^ have previously claimed that GDC is not required for standard scanners with gradients that are linear over a large FOV and where the head position is close to the isocenter. Most gradient coils have specifications of <1% linearity over a 40‐cm sphere.^27^ Nevertheless, in our study gradient distortion resulted in measurable cortical fallouts and displacements in the AFC, ITC, and POC. Gradient distortion is mild but global, so that it changes the volume size of whole EPI images. Therefore, the burden of whole image distortion cumulatively arises as registration errors in the volume periphery, where the AFC, ITC, and POC are located. Further, these cortices are located in a zone where gradient distortion was high enough for measurable cortical fallouts and displacements. As for the POC, the above‐mentioned structural factors might have also affected these quantities similarly.

Our results examining cortical fallout and displacement indicate that GDC is necessary for precise surface‐based fMRI analysis. Offline GDC is especially important when we use MRI scanners where the availability of a vendor's GDC depends on the scanning sequences. In fact, the vendor's GDC was not available in the present fMRI sequence on our 7T scanner, but it was available for the structural image sequences acquired at 3T. Conversely, the importance of offline GDC applications has been less emphasized in scanners where a vendor's GDC is applicable in fMRI sequences. However, replacing the vendor's GDC with our own offline one may be important, as the vendor's GDC is applied only in the in‐plane direction in 2D EPI sequences. Although comparison of 2D and 3D GDCs is outside the scope of this study, several studies have reported or referred to the effectiveness of 3D GDC over 2D GDC in structural imaging sequences,^17^, ^18^, ^36^ implying that offline GDC should not be confined to scanners that have a poor gradient nonlinearity due to a short or small bore and/or where the subject's head is not positioned at the isocenter.

The metrics used in our study (cortical fallout and displacement) were informative in demonstrating the impact of distortions on surface‐based analysis in functional neuroimaging. The introduction of cortical displacement highlighted a problem caused by voxels surviving on gray matter despite image distortion. The group‐averaged cortical displacement in the uncorrected condition was comparable to the length of a side of the voxel in this study (0.96 mm compared to 1.2 mm). The displacement ratio was also helpful to accentuate vertices where cortical displacement was observed. Meanwhile, because of the vertexwise metrics relying on only positional information of vertices, we did not consider partial cortical fallout resulting from voxel volume change and the partial volume effect. Moreover, a vertex was determined as a fallout when experiencing a distortion yielding displacement over half the cortical thickness in the direction normal to the surface. The present group‐averaged cortical fallout rates are, therefore, likely to be conservative. However, even if ~90% of the vertices could survive on gray matter without any distortion corrections in the conservative evaluation, fMRI data would be missampled on vertices with every surviving voxel displaced by nearly the length of the side of a voxel, on average.

### *Limitations*

We evaluated displacements only on a cortical surface modeled as a sheet. However, as the cortex indeed has thickness, displacements caused by image distortions should also be observed in the depth direction. These displacements are much more critical for cortical layer‐based analysis,^1^, ^8^, ^9^, ^10^ which is more advanced than surface‐based analysis.^37^, ^38^ For future study, we need to consider the reduction of image distortions during fMRI data acquisition and methods for more precise distortion corrections. At the same time, evaluation for displacements in the depth direction will be more important.

## Conclusion

This study quantified the effects of geometric distortion corrections in surface‐based analysis as cortical fallouts and displacements, rather than simple 3D Euclidean displacements, by creating cortical surfaces and ribbons replicating distortions of EPI images. Using these neurobiologically more meaningful quantities, our method of distortion correction evaluation revealed that even moderate distortion (B_0_ distortion in regions other than in the vicinity of the nasal cavity and ear canals and gradient distortion) can yield significant cortical fallouts and displacements in regions where the cortex is thin (eg, the primary somatosensory cortex), where the folding pattern of the cortex is complex (eg, the visual cortex), or where gradient linearity is imperfect (eg, the AFC, ITC, and POC, that is regions distant from the isocenter of an MRI scanner). This suggests that careful distortion corrections, as performed in the preprocessing pipelines, are essential for surface‐based analysis of high‐resolution fMRI data at 7T.

## References

[1] De Martino F , Moerel M , Ugurbil K , Goebel R , Yacoub E , Formisano E . Frequency preference and attention effects across cortical depths in the human primary auditory cortex. Proc Natl Acad Sci U S A 2015;112:16036‐16041. 26668397 10.1073/pnas.1507552112 PMC4702984

[2] Ogawa S , Tank DW , Menon R , et al. Intrinsic signal changes accompanying sensory stimulation: Functional brain mapping with magnetic resonance imaging. Proc Natl Acad Sci U S A 1992;89:5951‐5955. 1631079 10.1073/pnas.89.13.5951 PMC402116

[3] Yacoub E , Shmuel A , Pfeuffer J , et al. Imaging brain function in humans at 7 Tesla. Magn Reson Med 2001;45:588‐594. 11283986 10.1002/mrm.1080

[4] Vaughan JT , Garwood M , Collins CM , et al. 7T vs. 4T: RF power, homogeneity, and signal‐to‐noise comparison in head images. Magn Reson Med 2001;46:24‐30. 11443707 10.1002/mrm.1156

[5] Uğurbil K , Adriany G , Anderson P , et al. Ultrahigh field magnetic resonance imaging and spectroscopy. Magn Reson Imaging 2003;21:1263‐1281. 14725934 10.1016/j.mri.2003.08.027

[6] Chaimow D , Yacoub E , Uğurbil K , Shmuel A . Spatial specificity of the functional MRI blood oxygenation response relative to neuronal activity. NeuroImage 2018;164:32‐47. 28882632 10.1016/j.neuroimage.2017.08.077

[7] Yacoub E , Harel N , Uğurbil K . High‐field fMRI unveils orientation columns in humans. Proc Natl Acad Sci U S A 2008;105:10607‐10612. 18641121 10.1073/pnas.0804110105 PMC2492463

[8] Polimeni JR , Fischl B , Greve DN , Wald LL . Laminar analysis of 7T BOLD using an imposed spatial activation pattern in human V1. NeuroImage 2010;52:1334‐1346. 20460157 10.1016/j.neuroimage.2010.05.005 PMC3130346

[9] Muckli L , De Martino F , Vizioli L , et al. Contextual feedback to superficial layers of V1. Curr Biol 2015;25:2690‐2695. 26441356 10.1016/j.cub.2015.08.057 PMC4612466

[10] Kok P , Bains LJ , van Mourik T , Norris DG , de Lange FP . Selective activation of the deep layers of the human primary visual cortex by top‐down feedback. Curr Biol 2016;26:371‐376. 26832438 10.1016/j.cub.2015.12.038

[11] Polimeni JR , Renvall V , Zaretskaya N , Ficshl B . Analysis strategies for high‐resolution UHF‐fMRI data. NeuroImage 2018;168:296‐320. 28461062 10.1016/j.neuroimage.2017.04.053 PMC5664177

[12] Jezzard P , Balaban RS . Correction for geometric distortion in echo planar images from B_0_ field variations. Magn Reson Med 1995;34:65‐73. 7674900 10.1002/mrm.1910340111

[13] Hutton C , Bork A , Josephs O , Deichmann R , Ashburner J , Turner R . Image distortion correction in fMRI: A quantitative evaluation. NeuroImage 2002;16:217‐240. 11969330 10.1006/nimg.2001.1054

[14] Andersson JLR , Skare S , Ashburner J . How to correct susceptibility distortions in spin‐echo echo‐planar images: Application to diffusion tensor imaging. NeuroImage 2003;20:870‐888. 14568458 10.1016/S1053-8119(03)00336-7

[15] Robinson EC , Jbabdi S , Glasser MF , et al. MSM: A new flexible framework for multimodal surface matching. NeuroImage 2014;100:414‐426. 24939340 10.1016/j.neuroimage.2014.05.069 PMC4190319

[16] Smith SM , Jenkinson M , Woolrich MW , et al. Advances in functional and structural MR image analysis and implementation as FSL. NeuroImage 2004;23:S208‐S219. 15501092 10.1016/j.neuroimage.2004.07.051

[17] Janke A , Zhao H , Cowin GJ , Galloway GJ , Doddrell DM . Use of spherical harmonic deconvolution methods to compensate for nonlinear gradient effects on MRI images. Magn Reson Med 2004;52:115‐122. 15236374 10.1002/mrm.20122

[18] Jovicich J , Czanner S , Greve D , et al. Reliability in multi‐site structural MRI studies: Effects of gradient non‐linearity correction on phantom and human data. NeuroImage 2006;30:436‐443. 16300968 10.1016/j.neuroimage.2005.09.046

[19] Van Essen DC , Ugurbil K , Auerbach E , et al. The human connectome project: A data acquisition perspective. NeuroImage 2012;62:2222‐2231. 22366334 10.1016/j.neuroimage.2012.02.018 PMC3606888

[20] Glasser MF , Sotiropoulos SN , Wilson JA , et al. The minimal preprocessing pipelines for the human connectome project. NeuroImage 2013;80:105‐124. 23668970 10.1016/j.neuroimage.2013.04.127 PMC3720813

[21] Moeller S , Yacoub E , Olman CA , et al. Multiband multislice GE‐EPI at 7 Tesla, with 16‐fold acceleration using partial parallel imaging with application to high spatial and temporal whole‐brain fMRI. Magn Reson Med 2010;63:1144‐1153. 20432285 10.1002/mrm.22361 PMC2906244

[22] Griswold MA , Jakob PM , Heidemann RM , et al. Generalized autocalibrating partially parallel acquisitions (GRAPPA). Magn Reson Med 2002;47:1202‐1210. 12111967 10.1002/mrm.10171

[23] Mugler JP 3rd , Brookeman JR . Three‐dimensional magnetization‐prepared rapid gradient‐echo imaging (3D MP RAGE). Magn Reson Med 1990;15:152‐157. 2374495 10.1002/mrm.1910150117

[24] Mugler JP 3rd , Bao S , Mulkern RV , et al. Optimized single‐slab three‐dimensional spin‐echo MR imaging of the brain. Radiology 2000;216:891‐899. 10966728 10.1148/radiology.216.3.r00au46891

[25] Greve DN , Fischl B . Accurate and robust brain image alignment using boundary‐based registration. NeuroImage 2009;48:63‐72. 19573611 10.1016/j.neuroimage.2009.06.060 PMC2733527

[26] Glasser MF , Smith SM , Marcus DS , et al. The Human Connectome Project's neuroimaging approach. Nat Neurosci 2016;19:1175‐1187. 27571196 10.1038/nn.4361 PMC6172654

[27] Jezzard P , Clare S . Sources of distortion in functional MRI data. Hum Brain Mapp 1999;8:80‐85. 10524596 10.1002/(SICI)1097-0193(1999)8:2/3<80::AID-HBM2>3.0.CO;2-C PMC6873315

[28] Holland D , Kuperman JM , Dale AM . Efficient correction of inhomogeneous static magnetic field‐induced distortion in echo planar imaging. NeuroImage 2010;50:175‐183. 19944768 10.1016/j.neuroimage.2009.11.044 PMC2819607

[29] Smith SM , Beckmann CF , Andersson J , et al. Resting‐state fMRI in the Human Connectome Project. NeuroImage 2013;80:144‐168. 23702415 10.1016/j.neuroimage.2013.05.039 PMC3720828

[30] Togo H , Rokicki J , Yoshinaga K , et al. Effects of field‐map distortion correction on resting state functional connectivity MRI. Front Neurosci 2017;11:656. 29249930 10.3389/fnins.2017.00656 PMC5717028

[31] Fischl B , Dale AM . Measuring the thickness of the human cerebral cortex from magnetic resonance images. Proc Natl Acad Sci U S A 2000;97:11050‐11055. 10984517 10.1073/pnas.200033797 PMC27146

[32] MacDonald D , Kabani N , Avis D , Evans AC . Automated 3‐D extraction of inner and outer surfaces of cerebral cortex from MRI. NeuroImage 2000;12:340‐356. 10944416 10.1006/nimg.1999.0534

[33] Hutton C , De Vita E , Ashburner J , Deichmann R , Turner R . Voxel‐based cortical thickness measurements in MRI. NeuroImage 2008;40:1701‐1710. 18325790 10.1016/j.neuroimage.2008.01.027 PMC2330066

[34] Glasser MF , Van Essen DC . Mapping human cortical areas in vivo based on myelin content as revealed by T1‐ and T2‐weighted MRI. J Neurosci 2011;31:11597‐11616. 21832190 10.1523/JNEUROSCI.2180-11.2011 PMC3167149

[35] Rabiei H , Richard F , Coulon O , Lefèvre J . Local spectral analysis of the cerebral cortex: New gyrification indices. IEEE Trans Med Imaging 2017;36:838‐848. 27913336 10.1109/TMI.2016.2633393

[36] Wang D , Strugnell W , Cowin G , Doddrell DM , Slaughter R . Geometric distortion in clinical MRI systems part I: Evaluation using a 3D phantom. Magn Reson Imaging 2004;22:1211‐1221. 15607092 10.1016/j.mri.2004.08.012

[37] Dale AM , Fischl B , Sereno MI . Cortical surface‐based analysis. I. Segmentation and surface reconstruction. NeuroImage 1999;9:179‐194. 9931268 10.1006/nimg.1998.0395

[38] Fischl B , Sereno MI , Dale AM . Cortical surface‐based analysis. II: Inflation, flattening, and a surface‐based coordinate system. NeuroImage 1999;9:195‐207. 9931269 10.1006/nimg.1998.0396

